# Chemical Compositions of *Scutellaria* Essential Oils Cultivated in Eastern Oregon: *S. angustifolia*, *S. baicalensis*, *S. barbata*, and *S. lateriflora*

**DOI:** 10.3390/plants15071075

**Published:** 2026-04-01

**Authors:** Clinton C. Shock, Ambika Poudel, Prabodh Satyal, William N. Setzer

**Affiliations:** 1 Department of Crop and Soil Science, Oregon State University, Ontario, OR 97914, USA; 2 Aromatic Plant Research Center, 230 N 1200 E, Suite 100, Lehi, UT 84043, USApsatyal@aromaticplant.org (P.S.); 3 Department of Chemistry, University of Alabama in Huntsville, Huntsville, AL 35899, USA

**Keywords:** skullcap, Lamiaceae, gas chromatography, mass spectrometry, caryophyllene, germacrene D, 1-octen-3-ol

## Abstract

The genus *Scutellaria* (Lamiaceae) is a phytochemically rich and medicinally important group of plants. *Scutellaria* species have been characterized by biologically active flavonoids such as baicalin, baicalein, and wogonin. In the present study, the essential oils of *S. angustifolia*, *S. baicalensis*, *S. barbata*, and *S. lateriflora*, cultivated in eastern Oregon, were obtained by means of hydrodistillation and analyzed using gas chromatographic methods. We hypothesize that the essential oils have compositions that may play a role in the traditional uses and biological activities of the genus. The major components in *S. angustifolia* essential oils were germacrene D (32.5–58.3%), (*E*)-β-caryophyllene (4.9–29.2%), and β-bourbonene (2.8–9.4%). *Scutellaria barbata* essential oil was dominated by 1-octen-3-ol (59.9%), with lower concentrations of linalool (9.5%) and (2*E*)-hexenal (5.1%). The major components in the essential oil of *S. lateriflora* were 1-octen-3-ol (28.3%), acetophenone (24.8%), benzaldehyde (7.5%), limonene (6.0%), (*E*)-benzalacetone (5.9%), and β-phellandrene (5.1%). The major components of the essential oil of *S. baicalensis* were 1-octen-3-ol (22.3%), (*E*)-β-caryophyllene (22.3%), and germacrene D (28.3%). This study demonstrates that *Scutellaria* can be cultivated in eastern Oregon. Additionally, *S. angustifolia* essential oil has been characterized for the first time.

## 1. Introduction

The genus *Scutellaria* L. (Lamiaceae) comprises around 470 species of herbaceous plants found throughout the world [1]. The genus is chemically rich and therapeutically useful, largely due to flavonoid components [2,3]. *Scutellaria baicalensis* Georgi, in particular, has been investigated for anti-inflammatory, antioxidant, cytotoxic, neuroprotective, antimicrobial, and immunomodulatory effects, which have been attributed to flavones such as baicalin, baicalein, wogonin, and their glycosides [4,5,6]. In this work, we investigate whether important *Scutellaria* species can be cultivated in eastern Oregon. In addition, we hypothesize that the *Scutellaria* species synthesize volatile compounds that yield essential oils with compositions that may play a role in the traditional uses and biological activities of the genus. The purpose of this study is to examine the essential oil compositions of *Scutellaria* that have been successfully cultivated in eastern Oregon, which could highlight their potential utility as herbal medicines.

**Figure 1:**
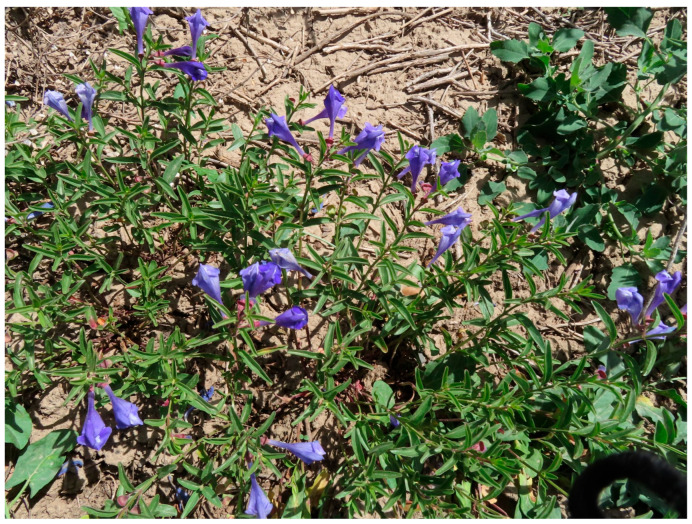
Photograph of *Scutellaria angustifolia* Pursh cultivated in Ontario, OR, USA.

*Scutellaria angustifolia* Pursh (narrowleaf skullcap, Lamiaceae) is native to western North America, including eastern Washington, eastern Oregon, Nevada, and Idaho [1,7,8]. The plant is perennial with single stems or stems branched near the bottom with stolons proliferating laterally underground; the leaves are gray–green, 5–45 mm long; the flowers are blue with a tube around 25 mm long [9] (Figure 1). There have apparently been no publications on the phytochemistry of this species.

*Scutellaria baicalensis* Georgi (Baikal skullcap, Lamiaceae) is a perennial rhizomatous herb native to east Asia, including China, Japan, Korea, Mongolia, and eastern Siberia [1,10]. The plant has a prominent history in Traditional Chinese Medicine (TCM) and is rich in flavonoids and other polyphenolic compounds, which have been extensively reviewed [4,5,6,11,12,13]. In addition, analyses of the essential oil from the aerial parts [14,15] and headspace volatiles from the flowers [16] of *S. baicalensis* have been reported.

*Scutellaria barbata* D. Don (barbed skullcap, Lamiaceae) is native to East Asia, principally southern China and the Korean Peninsula, but the plant is also reported in Taiwan, Japan, Laos, Myanmar, Thailand, and Vietnam [1,17]. Ethnobotanically, the plant is used in TCM and has shown hepatoprotective and anticancer properties [18]. The plant is a rich source of flavonoids [19,20,21] and diterpenoids [22,23,24,25,26,27], as well as antitumor polysaccharides [28,29]. The phytochemistry and pharmacology of *S. barbata* have been reviewed [30,31,32]. The essential oil compositions of *S. barbata* from Hubei, China [33], Hunan, China [34], and cultivated in south Alabama [14] have been reported.

*Scutellaria lateriflora* L. (blue skullcap, Lamiaceae) is indigenous to and widely distributed throughout North America. The plant has been a part of Native American (Cherokee) traditional medicine [35,36] as well as modern herbal medicine as a nervine and sedative [37,38]. The phytochemical profile of *S. lateriflora* shows the plant to be composed largely of flavonoids [39,40,41,42], diterpenoids [43], and amino acids [40]. The essential oil of *S. lateriflora*, cultivated in south Alabama, has been reported [14].

The four *Scutellaria* species in this study exhibit discernable morphological differences, which are summarized in Table 1.

## 2. Results

The *S. angustifolia* essential oils were obtained by means of hydrodistillation in yields of 5.40–9.17% as colorless, pale-yellow, or yellow oils. The *S. angustifolia* essential oils were dominated by sesquiterpene hydrocarbons (70.9–86.2%) with germacrene D (32.5–58.3%), (*E*)-β-caryophyllene (4.9–29.2%), and β-bourbonene (2.8–9.4%) as the major components (Table 2). The complete compositions are provided in Supplementary Table S1.

The essential oil compositions (major components) of *S. baicalensis*, *S. barbata*, and *S. lateriflora* are compiled in Table 3. The colorless essential oil of *S. baicalensis* was obtained in 6.70% yield and was rich in germacrene D (28.3%), 1-octen-3-ol (22.3%), and (*E*)-β-caryophyllene (22.3%). *Scutellaria barbata* essential oil was dominated by 1-octen-3-ol (59.9%) with lower concentrations of linalool (9.5%) and (2*E*)-hexenal (5.1%). The major components in the essential oil of *S. lateriflora* were 1-octen-3-ol (28.3%), acetophenone (24.8%), benzaldehyde (7.5%), limonene (6.0%), (*E*)-benzalacetone (5.9%), and β-phellandrene (5.1%). The complete compositions are provided in Supplementary Table S2.

## 3. Discussion

The essential oil of *S. baicalensis* in this study was dominated by 1-octen-3-ol (22.3%), (*E*)-β-caryophyllene (22.3%), and germacrene D (28.3%), which were also major components in *S. baicalensis* cultivated in south Alabama (6.1%, 11.6%, and 39.3%, respectively) [14]. In contrast, however, the south Alabama sample also had high concentrations of thymol (7.5%) and carvacrol (9.3%), which were not detected in the sample from eastern Oregon. The essential oil from the aerial parts of *S. baicalensis* collected from Tangshan, China, was also rich in (*E*)-β-caryophyllene (15.2%), and germacrene D (5.4%), as well as caryophyllene oxide (13.9%) and eugenol (18.4%); 1-octen-3-ol was not reported, however [15]. Eugenol was not observed in the cultivated sample from south Alabama but was a minor component (0.6%) in the sample from eastern Oregon in this study. A headspace volatile analysis of *S. baicalensis* flowers showed the floral volatiles to be dominated by sesquiterpene hydrocarbons, particularly (*E*)-β-caryophyllene (22.3–41.5%) and germacrene D (12.4–27.5%), but carvacrol, thymol, or 1-octen-3-ol were not reported [16]. The root essential oil of *S. baicalensis* was reported, but the constituents were not quantified [49]. Based on the gas chromatogram, the root oil had acetophenone, (*E*)-4-phenyl-2-butanone, palmitic acid, and oleic acid as major components; (*E*)-β-caryophyllene was detected, but in low concentration; and neither carvacrol nor thymol were detected. The current study adds to our knowledge of *S. baicalensis* phytochemistry, illustrating the variation in chemical composition.

(*E*)-β-Caryophyllene is well known for various biological activities. The compound has shown cytotoxic activity against several tumor-derived cell lines [50,51,52,53], antibacterial activity against several Gram-positive strains [50,52,53,54,55], and antifungal activity against *Candida albicans* [56]. (*E*)-β-Caryophyllene has also shown analgesic and anti-inflammatory activities [57,58,59,60,61]. Germacrene D has also demonstrated cytotoxicity against human tumor cell lines [50,62], antibacterial activity against Gram-positive organisms [55], and antifungal activity against *Aspergillus niger* [50]. Germacrene D has also exhibited immunomodulatory activity [63]. Thus, these sesquiterpene major components may contribute to the reported health benefits of *S. baicalensis*.

There is variation in the essential oil compositions of *S. barbata*, depending on the collection site (Table 4). The *S. barbata* essential oil from eastern Oregon was dominated by 1-octen-3-ol, which is a major component of essential oils from China and Alabama. Linalool was also found in all samples of *S. barbata* essential oil. Palmitic acid was abundant in essential oils from Hunan, China, and south Alabama, but was not observed in the sample from this study (eastern Oregon). Several factors may be responsible for variations in the chemical composition of essential oils within a species [64,65,66,67,68]. These include genetic factors [69,70,71], abiotic environmental characteristics of the collection sites [72,73], seasonality/phenology [74], and biotic factors such as herbivory [75,76] or fungal infection [77], as well as differences in processing methods. Interestingly, 1-octen-3-ol functions primarily as a volatile chemoattractant. The compound has been shown to be an attractant for hematophagous arthropods, including mosquitoes (*Aedes aegypti*, *Aedes albopictus*, *Culex quinquefasciatus*) [78,79], the tsetse fly *Glossina morsitans morsitans* [80], and the tick *Amblyomma americanum* [81].

The *S. lateriflora* essential oil composition in this study is qualitatively similar to the sample cultivated in south Alabama [14]. Both samples had high concentrations of 1-octen-3-ol (28.3% and 27.5%, respectively). While the concentration of acetophenone was high in the eastern Oregon sample (24.8%), it was lower in the south Alabama sample (3.6%). (*E*)-Benzalacetone concentrations were similar between the eastern Oregon and south Alabama samples (5.9% and 4.7%, respectively). Likewise, (*E*)-β-caryophyllene concentrations (3.9% and 8.8%, respectively) and benzaldehyde concentrations (7.5% and 2.0%, respectively) were comparable. The south Alabama sample had a high concentration of phytol (14.8%), but it was not observed in the eastern Oregon sample. Conversely, limonene (6.0% in the eastern Oregon sample) was not observed in the south Alabama sample.

To our knowledge, there have been no previous reports on the cultivation or essential oil composition of *S. angustifolia*. The essential oils in this study show the plant to be rich in sesquiterpene hydrocarbons, especially germacrene D (43.0 ± 7.7%), (*E*)-β-caryophyllene (15.0 ± 7.1%), and β-bourbonene (4.6 ± 1.7%), but relatively low in 1-octen-3-ol (2.3 ± 1.9%). Thus, *S. angustifolia* essential oil is similar in composition to *S. baicalensis*.

## 4. Materials and Methods

### 4.1. Plant Material

Plant materials of *Scutellaria angustifolia* were based on our original collections in nature from 12 distinct locations in western Idaho and eastern Oregon (Clinton and Candace Shock, Scientific Ecological Services, Ontario, OR, USA, 44.016225° N, 116.990308° W, 668 m elevation). The collection details are summarized in Table 5. Collections number 2 and 3 were *Scutellaria angustifolia* ssp. *micrantha* and the other ten collections were *Scutellaria angustifolia* ssp. *angustifolia.* The *S. angustifolia* samples were identified in the field by Clinton C. Shock. Pressed samples of the field specimens were verified by Richard Olmstead, Burke Museum Herbarium Curator (University of Washington). The collections were made by taking shallow stolons from the bases of 15 to 30 plants at each collection site. The collected stolons were grown into mature plants, and their stolons were subsequently used to establish field plots.

Planting materials for *Scutellaria baicalensis*, *S. barbata*, and *S. lateriflora* were selected and increased from plants previously selected in Ontario by Scientific Ecological Services. Plant selections were based on observed productivity and vigor. *Scutellaria baicalensis* and *S. barbata* transplants for the current study were grown from seed while *S. lateriflora* transplants were grown from stolons and seed.

Plants of all four *Scutellaria* species for the current study were planted in silt loam soil in Ontario, Oregon, by Scientific Ecological Services, Ontario, OR, USA. The field for planting had been disked, rototilled, and bedded into plots 3 m wide and 45 or 90 m long. Each plot consisted of four 75-cm beds. Drip tape was shanked into the soil at 10 cm depth in every bed. The drip tape (Dripnet PC, Netafim, Fresno, CA, USA) had emitters spaced 30 cm apart and an emitter flow rate of 1.9 L/h at 69 kPa. Transplants were planted at 30 cm spacing down each bed. Areas for each of the 12 *Scutellaria angustifolia* selections were established in the two 90-m plots and the other three species were planted separately in three 45-m plots. No preplant fertilizer was applied.

Plants were irrigated with drip irrigation 1 or 2 times per week for 6 h. During the first two weeks, plants received 2 irrigations per week and only 1 irrigation per week during most of the rest of the growing season. The crop was fertilized through the drip irrigation system, twice with 12 kg/ha of N as urea ammonium nitrate and once with 0.07 kg/ha of Fe as iron EDDTA.

Aerial parts of all plants were harvested at full bloom in 2025. The top 10 cm of flowering branches of *Scutellaria angustifolia* selections were clipped on 25 May or 29 May; samples #11 and #12 were recollected on 8 August. The top 30 cm of the flowering branches of *Scutellaria baicalensis*, *S. barbata*, and *S. lateriflora* were trimmed on 15 July, 29 May, and 15 July, respectively. Trimmed flowering branches were immediately frozen.

### 4.2. Hydrodistillation

For each *Scutellaria* plant sample, the fresh/frozen aerial parts were chopped and hydrodistilled using a Likens–Nickerson apparatus [82,83,84] for 4 h with continuous extraction of the distillate with dichloromethane to give the essential oils (Table 6). The plant material was added to a 1000-mL flask along with enough distilled water to cover the plant material. The dichloromethane (20 mL) was added to the receiving flask. The dichloromethane was evaporated using a stream of dry air to give the essential oils.

### 4.3. Gas Chromatographic Analysis

The *Scutellaria* essential oils were analyzed by means of gas chromatography—mass spectrometry (GC-MS) and gas chromatography with flame ionization detection (GC-FID) as reported previously [85]. Instrument details are provided in Supplementary Table S3. Retention index values were calculated using the arithmetic formula of van den Dool and Kratz [44]. Essential oil components were identified through comparison of their retention index values (within 10 RI units) and their mass spectral fragmentation (> 80% MS match) with those reported in the databases of Adams [45], Satyal [46], Mondello [47], and NIST20 [48]. Percentages of the essential oil components were calculated based on peak integration without standardization.

## 5. Conclusions

Based on this study, *Scutellaria* species (*S. angustifolia*, *S. baicalensis*, *S. barbata*, and *S. lateriflora*) can be successfully cultivated in eastern Oregon. The essential oils of *S. angustifolia* and *S. baicalensis* are rich in sesquiterpene hydrocarbons, particularly germacrene D and (*E*)-β-caryophyllene. The essential oil compositions of *S. baicalensis* and *S. barbata* show wide variation depending on geographical location, which highlight the potential importance of environmental factors in essential oil profiles, although genetic differences in the plant materials cannot be ruled out. Additional studies on *S. baicalensis* and *S. barbata* from other geographical locations are needed to verify the variability of essential oil compositions. Major sesquiterpene components such as germacrene D and (*E*)-β-caryophyllene are known to exhibit cytotoxic, antibacterial, antifungal, analgesic, and anti-inflammatory activities, which likely contribute to the therapeutic benefits of *Scutellaria* species. This study provides the first report on the essential oil composition of *S. angustifolia*, expanding our understanding of the phytochemistry of the *Scutellaria* genus. Nevertheless, there are numerous unstudied and understudied *Scutellaria* species that should be examined to define the phytochemistry of this genus. In addition, further studies of the volatile chemical profiles of cultivated *Scutellaria*, repeated annually over several seasons, should be carried out.

## Figures and Tables

**Table 1:** Morphological differences between the four *Scutellaria* species cultivated in Ontario, Oregon.

Species	Narrowleaf Skullcap (*Scutellaria angustifolia*)	Blue Skullcap (*Scutellaria lateriflora*)	Baikal Skullcap (*Scutellaria baicalensis*)	Barbat Skullcap (*Scutellaria barbata*)
Naturally occurring region	North America intermountain west (dry slopes)	North America (wetlands)	Siberia, Mongolia, N. China (dry slopes)	South–Central and SE China (moist fields)
Plant structure and growth habit	Perennial growing from prolific underground stolons, thin square stems with little or no branching, 5 to 20 cm tall, ours 10–30 cm	Perennial growing from underground stolons, square stems with prolific branching, 60–90 cm tall, ours 60–100 cm	Perennial with square stems growing from a woody base, stems with prolific branching, 20 to 30 cm tall, ours 50–80 cm	Perennial with thin square stems growing from a compact base, many stems with little or no branching, 20 to 30 cm tall, ours 50–75 cm
Leaves	Smooth lanceolate to ovate leaves, ours to 21 by 10 mm	Thin, ovate, lightly toothed leaves, ours to 61 by 29 mm	Narrow, lance-shaped leaves, ours to 42 by 11 mm	Triangular–lanceolate leaves, ours to 32 by 17 mm
Flowers	Intense blue, violet, or blue and white flowers, 14–27 mm by 6–11 mm	Small blue–violet or light blue flowers borne on one-sided racemes, 7 mm by 2.5–3 mm	Purple–violet or intense blue in upright spikes, ours 22–25 by 9–11 mm	Violet–blue to light blue, tiny, paired blooms, 14–15 mm by 5 mm
Plant part used medicinally	Leaves and stems by herbalists	Leaves and stems by herbalists	Root in traditional Chinese medicine	Whole plant or aerial parts in traditional Chinese medicine

**Table 2:** Major chemical components of *Scutellaria angustifolia* essential oils cultivated in Ontario, Oregon.

RI_calc_	RI_db_	Compounds	#1	#2	#3	#4	#5	#6	#7	#8	#10	#11	#11 (8-8)	#12	#12 (8-8)
850	850	(2*E*)-Hexenal	1.3	0.2	3.1	3.3	2.0	0.6	1.3	6.2	1.1	2.8	3.2	1.1	2.5
962	960	Benzaldehyde	1.8	0.2	2.2	2.3	2.2	-	0.9	1.6	0.8	0.6	1.7	0.8	1.3
977	974	1-Octen-3-ol	2.3	0.6	6.0	2.1	1.7	0.1	-	3.5	0.1	1.6	5.3	1.1	2.8
1097	1099	Linalool	1.1	3.1	0.9	4.9	3.6	4.3	2.2	1.2	2.1	6.8	1.7	7.7	0.7
1359	1356	(*E*)-Benzalacetone	2.6	0.2	3.9	4.2	3.7	-	2.8	1.2	-	0.8	2.8	1.1	1.6
1374	1377	α-Copaene	1.2	1.3	1.2	1.4	1.4	2.6	1.9	1.3	2.1	1.8	1.1	1.5	1.5
1382	1382	β-Bourbonene	2.8	3.1	3.2	4.0	4.3	6.1	4.5	5.4	9.4	4.9	3.4	4.4	4.5
1389	1390	*trans*-β-Elemene	1.1	1.1	1.1	1.2	1.3	1.8	1.5	0.9	1.8	1.7	1.1	1.8	2.3
1420	1417	(*E*)-β-Caryophyllene	25.0	29.2	18.1	11.7	12.8	15.6	10.5	21.6	12.4	7.6	18.1	8.1	4.9
1430	1433	β-Copaene	1.1	0.7	0.8	0.9	1.0	1.2	1.2	1.0	2.2	1.1	0.9	1.0	1.1
1452	1452	(*E*)-β-Farnesene	1.5	1.2	1.5	1.4	1.6	-	1.6	0.8	0.9	1.8	1.1	2.0	0.5
1456	1454	α-Humulene	2.6	2.9	2.1	1.7	1.9	2.1	1.6	2.0	1.8	1.8	2.0	1.9	1.2
1481	1480	Germacrene D	32.5	41.6	40.5	42.9	46.5	48.8	43.8	29.4	38.9	48.8	37.2	50.1	58.3
1517	1518	δ-Cadinene	1.7	1.3	1.4	1.6	1.3	1.6	3.1	1.3	2.3	1.5	2.1	1.6	1.2
1658	1655	α-Cadinol	0.8	2.4	1.9	2.2	2.0	3.2	4.1	1.7	4.3	3.0	3.6	2.5	1.4

RI_calc_ = Retention index determined with respect to a homologous series of *n*-alkanes on a ZB-5ms column using the method of van den Dool and Kratz [44]. RI_db_ = Reference retention index obtained from the databases [45,46,47,48].

**Table 3:** Major chemical components of *Scutellaria baicalensis*, *Scutellaria barbata*, and *Scutellaria lateriflora* essential oils cultivated in Ontario, Oregon.

RI_calc_	RI_db_	Compound	*S. baicalensis*	*S. barbata*	*S. lateriflora*
802	801	Hexanal	0.1	2.0	1.3
849	849	(2*E*)-Hexenal	0.6	5.1	3.4
851	853	(3Z)-Hexenol	0.5	4.4	1.6
961	960	Benzaldehyde	-	-	7.5
978	978	1-Octen-3-ol	22.3	59.9	28.3
995	996	3-Octanol	2.4	3.1	-
1030	1030	Limonene	tr	-	6.0
1031	1031	β-Phellandrene	-	-	5.1
1044	1043	Phenylacetaldehyde	0.2	1.3	0.7
1044	1044	Salicylaldehyde	-	-	1.0
1064	1064	Acetophenone	0.1	-	24.8
1098	1099	Linalool	4.1	9.5	1.2
1286	1290	*o*-Acetanisole	-	-	2.8
1356	1356	(*E*)-Benzalacetone	-	-	5.9
1382	1382	β-Bourbonene	2.0	1.2	-
1420	1417	(*E*)-β-Caryophyllene	22.3	3.1	3.9
1455	1454	α-Humulene	2.0	0.5	1.4
1481	1480	Germacrene D	28.3	4.8	-
1496	1497	Bicyclogermacrene	3.2	0.7	-
1518	1518	δ-Cadinene	1.2	0.5	-
1645	1645	τ-Muurolol	1.8	-	-
2144	2143	Serratol	-	1.2	-
2500	2500	Pentacosane	-	1.4	-

RI_calc_ = Retention index determined with respect to a homologous series of *n*-alkanes on a ZB-5ms column using the method of van den Dool and Kratz [44]. RI_db_ = Reference retention index obtained from the databases [45,46,47,48]. tr = trace (<0.05%).

**Table 4:** Comparison of major essential oil components of *Scutellaria barbata*.

Compounds	Collection Site				
	Hubei, China ^a^	Hunan, China ^b^	Newville, Alabama ^c^		Ontario, Oregon ^d^
1-Octen-3-ol	7.1	6.2	25.6	20.1	59.9
Linalool	6.7	5.8	3.3	2.5	9.5
Menthol	7.7	-	-	-	-
Thymol	1.4	-	2.2	7.7	-
Carvacrol	-	-	2.3	8.9	-
Methyl eugenol	5.6	1.2	-	-	-
(*E*)-β-Caryophyllene	-	4.4	3.6	2.7	3.1
(*Z*)-α-trans-Bergamotol	5.1	-	-	-	-
Phytone	11.0	4.6	-	0.6	-
1-Heptadecanol	5.0	-	-	-	-
Palmitic acid	-	28.6	15.6	13.0	-
Phytol	7.8	-	1.8	2.3	-

^a^ Yu et al., 2004 [33]; ^b^ Pan et al., 2011 [34]; ^c^ Lawson et al., 2021 [14]; ^d^ this work.

**Table 5:** Collection details for *Scutellaria angustifolia* samples collected in eastern Oregon and western Idaho.

Selection Number	Collection Site	Collection Date	Coordinates		Elevation (m)
			North	West	
1	West of Riggins, Idaho	26 June 2018	45.552	116.404	1585
2	Reynolds Creek, Idaho	18 April 2020	43.260	116.784	1225
3	Brogan Hill, Oregon	21 April 2020	44.264	117.624	1041
4	Cow Creek, Lucille, Idaho	26 April 2020	45.628	116.411	1929
5	Mitchell, Oregon	17 May 2020	44.561	120.059	1292
6	Fairview Campground, Oregon	18 May 2020	44.955	119.715	1289
7	Grids Creek, Oregon	18 May 2020	44.708	120.172	623
8	Austin, Oregon	19 May 2020	44.586	118.442	1298
9	Twinkenham, Oregon	31 May 2020	44.824	120.153	764
10	Fossil, Oregon	31 May 2020	44.895	120.108	1079
11	Clarno, Oregon	1 June 2020	44.901	120.324	648
12	Southwest of McCall, Idaho	21 May 2021	44.953	116.186	1566

**Table 6:** Hydrodistillation details for *Scutellaria* samples cultivated in Ontario, OR.

Sample	Mass Plant Material (g)	Mass Essential Oil (g)	% Yield	Color
*Scutellaria angustifolia* #1	61.55	4.0438	6.570	pale yellow
*Scutellaria angustifolia* #2	109.96	6.0023	5.459	pale yellow
*Scutellaria angustifolia* #3	65.55	4.2037	6.413	pale yellow
*Scutellaria angustifolia* #4	53.46	3.4358	6.427	yellow
*Scutellaria angustifolia* #5	48.33	4.2186	8.729	pale yellow
*Scutellaria angustifolia* #6	46.75	3.7603	8.043	pale yellow
*Scutellaria angustifolia* #7	61.55	4.2111	6.842	pale yellow
*Scutellaria angustifolia* #8	75.76	5.0012	6.601	pale yellow
*Scutellaria angustifolia* #10	46.54	4.2695	9.174	yellow
*Scutellaria angustifolia* #11	99.21	5.3532	5.396	pale yellow
*Scutellaria angustifolia* #11 (8/8)	69.59	4.1792	6.005	colorless
*Scutellaria angustifolia* #12	73.63	4.5978	6.244	pale yellow
*Scutellaria angustifolia* #12 (8/8)	52.82	3.7001	7.005	colorless
*Scutellaria baicalensis*	84.33	5.6537	6.704	colorless
*Scutellaria barbata*	44.91	5.2731	11.741	colorless
*Scutellaria lateriflora*	93.65	5.1872	5.539	pale yellow

## Data Availability

All data are available in this report. Additional information is available from the corresponding author upon reasonable request.

## Supplementary Materials

The following supporting information can be downloaded at https://www.mdpi.com/article/10.3390/plants15071075/s1. Table S1. Chemical compositions of *Scutellaria angustifolia* essential oils cultivated in Ontario, Oregon, Table S2. Chemical compositions of *Scutellaria baicalensis*, *Scutellaria barbata*, and *Scutellaria lateriflora* essential oils cultivated in Ontario, Oregon, Table S3. Instrument details for the gas chromatographic analyses of *Scutellaria* species cultivated in Ontario, Oregon.
