# Supplementary material for: Chemical Compositions of Scutellaria Essential Oils Cultivated in Eastern Oregon: S. angustifolia, S. baicalensis, S. barbata, and S. lateriflora
Source: Plants (Basel). 2026 Apr 1;15(7):1075. doi: 10.3390/plants15071075 (PMC13074248; doi:10.3390/plants15071075)
Supplement: Supplementary file 1 [file plants-15-01075-s001.zip › plants-4179872-supplementary.pdf]

Supplementary Table S1. Chemical compositions of *Scutellaria angustifolia* essential oils cultivated in Ontario, Oregon.

RI$_{calc}$	RI$_{db}$	Compounds	#1	#2	#3	#4	#5	#6	#7	#8	#10	#11	#11(8-8)	#12	#12(8-8)
796	797	(3*Z*)-Hexenal	-	-	0.1	-	-	0.2	-	0.1	0.3	-	0.1	-	0.1
801	801	Hexanal	0.3	0.1	0.5	0.5	0.3	0.1	0.2	1.0	0.2	0.3	0.4	0.2	0.4
850	850	(2*E*)-Hexenal	1.3	0.2	3.1	3.3	2.0	0.6	1.3	6.2	1.1	2.8	3.2	1.1	2.5
853	853	(3*Z*)-Hexenol	-	0.2	1.2	0.6	0.7	-	-	0.7	-	0.1	0.6	0.2	0.9
891	891	Styrene	0.3	-	0.2	0.2	0.1	-	0.1	0.2	0.1	-	0.2	0.1	0.1
901	901	Heptanal	-	-	-	0.1	0.2	0.1	-	-	-	0.1	-	0.1	0.2
933	933	α-Pinene	-	-	-	-	-	-	-	0.1	-	-	0.1	-	-
962	960	Benzaldehyde	1.8	0.2	2.2	2.3	2.2	-	0.9	1.6	0.8	0.6	1.7	0.8	1.3
977	974	1-Octen-3-ol	2.3	0.6	6.0	2.1	1.7	0.1	-	3.5	0.1	1.6	5.3	1.1	2.8
996	996	3-Octanol	-	0.2	0.1	0.4	0.1	-	-	-	-	-	-	0.1	-
1009	1009	δ-3-Carene	-	-	-	-	-	-	-	0.2	-	-	0.1	-	-
1028	1030	Limonene	-	-	-	-	-	0.1	-	-	0.1	0.3	-	-	0.4
1030	1031	β-Phellandrene	-	-	-	-	-	-	-	-	-	0.3	-	-	0.3
1044	1043	Phenylacetaldehyde	0.4	1.3	0.6	1.5	1.7	-	1.1	0.4	0.3	0.7	0.7	0.9	0.6
1065	1064	Acetophenone	1.9	0.2	1.7	0.9	0.6	-	0.4	1.4	-	0.2	1.3	0.3	0.8
1097	1099	Linalool	1.1	3.1	0.9	4.9	3.6	4.3	2.2	1.2	2.1	6.8	1.7	7.7	0.7
1104	1104	Nonanal	0.2	0.1	0.1	-	0.2	-	0.1	-	-	0.1	-	0.1	0.1
1181	1180	Terpinen-4-ol	-	-	-	-	-	-	-	-	0.1	-	-	-	-
1194	1195	α-Terpineol	0.4	-	-	-	-	-	-	-	0.2	-	1.1	-	-
1287	1287	Dihydroedulan IA	-	0.2	-	-	-	-	-	0.2	-	-	-	-	-
1323	1322	Methyl decanoate	0.8	-	-	-	-	-	-	-	-	-	-	-	-
1344	1346	α-Cubebene	0.2	0.2	0.1	-	0.1	0.2	0.3	0.2	0.3	0.4	0.2	0.3	0.8
1345	1348	α-Longipinene	-	-	-	-	-	-	-	-	0.1	-	-	-	-
1353	1357	Eugenol	-	-	0.2	0.3	0.2	0.6	-	-	-	0.8	0.8	0.6	0.8
1359	1356	(*E*)-Benzalacetone	2.6	0.2	3.9	4.2	3.7	-	2.8	1.2	-	0.8	2.8	1.1	1.6
1364	1371	α-Ylangene	-	-	-	-	-	-	-	-	0.1	-	-	-	-
1374	1377	α-Copaene	1.2	1.3	1.2	1.4	1.4	2.6	1.9	1.3	2.1	1.8	1.1	1.5	1.5
1381	1383	*cis*-β-Elemene	-	-	-	-	-	-	0.1	0.3	0.3	-	-	0.1	tr
1382	1382	β-Bourbonene	2.8	3.1	3.2	4.0	4.3	6.1	4.5	5.4	9.4	4.9	3.4	4.4	4.5

1385	1385	α-Bourbonene	-	0.1	-	-	-	0.6	-	0.3	0.6	-	0.3	0.2	-
1386	1387	β-Cubebene	0.5	0.3	0.5	0.4	0.5	0.8	0.5	0.2	0.8	0.9	-	0.7	1.5
1389	1390	*trans*-β-Elemene	1.1	1.1	1.1	1.2	1.3	1.8	1.5	0.9	1.8	1.7	1.1	1.8	2.3
1413	1413	α-Barbatene	0.3	-	-	-	-	-	-	0.2	-	-	-	-	-
1418	1422	β-Ylangene	0.9	0.7	0.8	0.8	0.9	1.0	1.1	0.9	1.6	0.9	0.9	1.0	0.8
1420	1417	(*E*)-β-Caryophyllene	25.0	29.2	18.1	11.7	12.8	15.6	10.5	21.6	12.4	7.6	18.1	8.1	4.9
1430	1433	β-Copaene	1.1	0.7	0.8	0.9	1.0	1.2	1.2	1.0	2.2	1.1	0.9	1.0	1.1
1432	1432	*trans*-α-Bergamotene	0.5	0.3	0.3	0.5	0.4	-	0.3	0.5	0.7	0.5	0.4	0.4	0.2
1433	1433	*cis*-Thujopsene	0.1	-	-	-	-	-	-	0.3	-	-	-	-	-
1442	1438	Aromadendrene	0.2	0.1	0.2	0.2	0.2	0.3	0.3	0.2	0.5	0.3	0.2	0.3	0.2
1446	1446	*cis*-Muurola-3,5-diene	0.3	-	-	-	-	-	-	-	-	-	-	-	-
1449	1448	*trans*-Muurola-3,5-diene	0.2	-	-	-	-	-	-	-	0.1	-	-	-	-
1452	1452	(*E*)-β-Farnesene	1.5	1.2	1.5	1.4	1.6	-	1.6	0.8	0.9	1.8	1.1	2.0	0.5
1456	1454	α-Humulene	2.6	2.9	2.1	1.7	1.9	2.1	1.6	2.0	1.8	1.8	2.0	1.9	1.2
1461	1463	*cis*-Muurola-4(14),5-diene	1.4	-	-	-	-	0.4	1.1	0.5	0.3	0.4	-	0.1	-
1468	1464	9-*epi*-(*E*)-Caryophyllene	0.4	0.3	0.4	0.4	0.4	0.6	0.6	0.4	0.8	0.4	0.4	0.4	0.3
1472	1472	Cadina-1(6),4-diene	0.1	-	-	-	-	-	-	-	-	-	-	-	-
1475	1478	γ-Muurolene	0.5	0.3	0.3	0.4	0.3	0.7	0.6	0.3	1.4	0.4	0.5	0.5	0.4
1479	1479	α-Amorphene	-	-	-	-	-	-	0.2	-	0.3	-	-	-	-
1481	1480	Germacrene D	32.5	41.6	40.5	42.9	46.5	48.8	43.8	29.4	38.9	48.8	37.2	50.1	58.3
1482	1482	γ-Himachalene	-	0.5	0.6	0.6	0.6	-	0.5	0.6	-	0.6	0.5	0.5	0.8
1492	1492	*trans*-Muurola-4(14),5-diene	0.9	0.1	0.1	0.1	-	0.3	0.9	0.5	0.7	0.4	0.2	0.3	0.2
1493	1495	(*E*)-Methyl isoeugenol	-	0.1	-	0.2	-	-	0.9	-	-	0.4	-	0.3	-
1495	1497	Valencene	0.4	-	-	-	-	-	0.2	0.3	-	-	-	-	-
1496	1497	α-Selinene	-	-	-	-	-	-	-	-	-	-	-	0.1	-
1498	1497	α-Muurolene	0.6	0.3	0.5	0.3	0.3	0.6	0.7	0.3	1.3	0.5	0.5	0.5	0.4
1501	1501	Epizonarene	-	-	0.2	-	-	-	0.2	0.3	0.2	0.1	0.2	0.1	-
1503	1503	(*E*,*E*)-α-Farnesene	0.2	-	-	-	-	-	-	-	-	-	-	-	-
1506	1506	α-Chamigrene	0.2	-	-	-	-	-	-	-	-	-	-	-	-
1513	1512	γ-Cadinene	0.9	0.3	0.3	0.3	0.1	0.8	0.8	0.4	1.2	0.4	0.4	0.3	0.3
1517	1518	δ-Cadinene	1.7	1.3	1.4	1.6	1.3	1.6	3.1	1.3	2.3	1.5	2.1	1.6	1.2

1521	1521	Zonarene	0.3	-	-	-	-	-	0.3	-	0.1	-	-	-	-
1526	1528	(*E*)-γ-Bisabolene	0.3	-	-	-	-	-	-	0.1	-	-	0.1	-	-
1532	1533	*trans*-Cadina-1,4-diene	0.3	-	-	-	-	-	0.1	-	0.2	-	-	0.1	-
1533	1535	γ-Cuprenene	-	-	-	-	-	-	-	0.1	-	-	-	-	-
1537	1538	α-Cadinene	0.4	0.1	0.1	-	-	-	0.3	0.1	0.3	0.1	0.1	0.1	-
1562	1560	1-*nor*-Bourbanol	-	-	-	-	-	-	-	-	0.1	-	-	-	-
1564	1564	β-Calacorene	-	-	-	-	-	-	-	-	0.1	-	-	-	-
1569	1566	1,5-Epoxysalvial-4(14)ene	-	-	-	-	-	-	-	-	0.1	-	-	-	-
1576	1574	Germacra-1(10),5-dien-4β-ol	-	1.2	0.9	0.8	1.5	0.6	-	-	0.3	0.1	0.4	0.5	0.3
1581	1587	Caryophyllene oxide	-	0.7	0.5	0.2	0.3	0.4	0.3	0.9	0.6	0.5	0.7	0.3	0.2
1594	1596	Salvial-4(14)-en-1-one	-	-	-	-	-	-	-	-	0.3	-	-	-	-
1602	1600	α-Oplopenone	-	0.1	-	-	-	-	-	-	-	-	-	-	-
1612	1613	Humulene epoxide II	-	-	-	-	-	-	-	-	0.1	-	-	-	-
1617	1616	1,10-di-*epi*-Cubenol	-	-	-	-	-	-	-	-	0.1	-	-	-	-
1630	1631	1-*epi*-Cubenol	-	-	-	-	-	-	-	-	0.1	-	-	-	-
1643	1645	(*E*)-*iso*-Elemicin	-	-	-	-	-	-	-	-	-	-	-	0.1	-
1644	1643	τ-Cadinol	0.2	0.4	0.3	0.6	0.4	0.1	0.5	0.2	-	0.1	0.9	0.3	0.3
1645	1643	Cubenol	-	-	-	-	-	-	-	-	0.2	-	-	-	-
1646	1645	τ-Muurolol	0.2	0.9	0.4	0.9	0.8	1.1	1.9	0.4	2.1	1.3	1.2	0.7	0.8
1658	1655	α-Cadinol	0.8	2.4	1.9	2.2	2.0	3.2	4.1	1.7	4.3	3.0	3.6	2.5	1.4
1739	1738	(*E*)-Sesquilavandulyl acetate	-	0.5	-	-	-	-	-	-	-	-	-	-	-
1740	1735	Mint sulfide	-	-	-	-	-	-	-	-	0.2	0.1	0.1	0.1	0.1
1833	1835	1-Phytadiene	-	-	-	0.3	0.2	-	0.9	0.2	-	0.4	0.3	0.6	0.2
1839	1841	Phytone	0.3	-	-	0.3	0.2	-	0.3	0.2	-	-	-	0.1	0.2
1858	1861	3-Phytadiene	-	-	-	-	-	-	0.3	-	-	0.1	-	0.2	-
1876	1879	4-Phytadiene	-	-	-	-	-	-	0.4	-	-	0.2	-	0.2	0.1
1951	1951	(3*E*)-Cembrene A	0.3	-	-	-	-	-	-	-	-	-	-	-	-
1967	1968	Kaur-15-ene	-	-	-	-	-	-	-	4.0	-	-	-	-	-
2017	2015	Kaur-16-ene	-	-	-	-	-	-	-	2.3	-	-	-	-	-
2048	2049	Abietatriene	-	-	-	-	-	-	-	0.7	-	-	-	-	-
2078	2064	(9*Z*)-Octadecenenitrile	0.5	-	-	-	-	-	-	-	-	-	-	-	-

2145	2143	Serratol	1.3	-	-	-	-	-	-	-	-	-	-	-	-
2300	2300	Tricosane	0.2	-	0.1	0.4	0.2	-	0.4	-	-	0.1	-	0.1	0.2
2500	2500	Pentacosane	0.6	0.1	0.2	0.9	0.6	-	0.6	-	-	0.2	0.3	0.2	0.3
2700	2700	Heptacosane	0.7	0.1	0.2	0.8	0.5	-	0.6	-	-	-	-	0.1	0.3
		Monoterpene hydrocarbons	0.0	0.0	0.0	0.0	0.0	0.1	0.0	0.3	0.1	0.6	0.2	0.0	0.7
		Oxygenated monoterpenoids	1.5	3.1	0.9	4.9	3.6	4.3	2.2	1.2	2.4	6.8	2.8	7.7	0.7
		Sesquiterpene hydrocarbons	79.7	85.9	74.2	71.0	75.8	86.2	78.9	70.9	83.7	77.2	72.0	78.6	81.5
		Oxygenated sesquiterpenoids	1.2	6.2	3.9	4.7	5.0	5.5	6.7	3.2	8.2	5.1	6.8	4.3	3.0
		Diterpenoids	1.6	0.0	0.0	0.3	0.2	0.0	1.6	7.2	0.0	0.7	0.3	1.0	0.3
		Benzenoid aromatics	6.9	2.0	8.9	9.5	8.5	0.6	6.2	4.8	1.2	3.4	7.5	4.2	5.2
		Fatty acid derivatives	5.3	1.4	11.0	7.1	5.1	1.1	1.6	11.5	1.8	5.0	9.6	2.8	6.9
		n-Alkanes	1.5	0.2	0.6	2.2	1.3	0.0	1.6	0.0	0.0	0.3	0.3	0.4	0.8
		Others	0.3	0.2	0.0	0.3	0.2	0.0	0.3	0.4	0.3	0.1	0.1	0.1	0.3
		Total identified	*98.2*	*98.9*	*99.6*	*100.0*	*99.7*	*97.7*	*99.1*	*99.5*	*97.6*	*99.1*	*99.7*	*99.1*	*99.5*

RI_{calc} = Retention index determined with respect to a homologous series of *n*-alkanes on a ZB-5ms column using the method of van den Dool and Kratz [44]. RI_{db} = Reference retention index obtained from the databases [45–48].

Supplementary Table S2. Chemical compositions of *Scutellaria baicalensis*, *Scutellaria barbata*, and *Scutellaria lateriflora* essential oils cultivated in Ontario, Oregon.

RI_{calc}	RI_{db}	Compound	*S. baicalensis*	*S. barbata*	*S. lateriflora*
796	797	(3*Z*)-Hexenal	-	-	0.3
802	801	Hexanal	0.1	2.0	1.3
849	849	(2*E*)-Hexenal	0.6	5.1	3.4
851	853	(3Z)-Hexenol	0.5	4.4	1.6
891	891	Styrene	-	-	0.6
961	960	Benzaldehyde	-	-	7.5
975	973	1-Octen-3-one	0.1	0.7	0.1
978	978	1-Octen-3-ol	22.3	59.9	28.3
986	898	Myrcene	-	-	0.4
995	996	3-Octanol	2.4	3.1	-
1025	1025	*p*-Cymene	-	-	0.1
1030	1030	Limonene	tr	-	6.0
1031	1031	β-Phellandrene	-	-	5.1
1044	1043	Phenylacetaldehyde	0.2	1.3	0.7
1044	1044	Salicylaldehyde	-	-	1.0
1064	1064	Acetophenone	0.1	-	24.8
1098	1099	Linalool	4.1	9.5	1.2
1105	1107	Nonanal	-	-	0.2
1160	1161	2′-Hydroxyacetophenone	-	-	0.3
1194	1195	α-Terpineol	0.3	-	-
1286	1290	*o*-Acetanisole	-	-	2.8
1335	1335	δ-Elemene	0.2	-	-
1349	1348	α-Longipinene	-	-	0.4
1354	1356	Eugenol	0.6	-	-
1356	1356	(*E*)-Benzalacetone	-	-	5.9
1377	1377	α-Copaene	0.1	-	-
1382	1382	β-Bourbonene	2.0	1.2	-
1383	1380	2-*epi*-α-Funebrene	0.1	-	-
1388	1385	α-Bourbonene	0.1	-	-
1389	1387	β-Cubebene	tr	-	-
1390	1390	*trans*-β-Elemene	0.1	-	-
1405	1405	α-Funebrene	0.1	-	-
1407	1405	(*Z*)-β-Caryophyllene	-	-	0.4
1417	1414	α-Cedrene	tr	-	-
1419	1422	β-Ylangene	0.4	-	-
1420	1417	(*E*)-β-Caryophyllene	22.3	3.1	3.9
1423	1428	β-Duprezianene	0.1	-	-
1425	1421	β-Cedrene	0.1	-	-
1430	1430	β-Copaene	0.5	0.2	-

1433	1433	Coumarin	-	-	0.3
1447	1447	*iso*-Germacrene D	0.2	-	-
1447	1447	β-Barbatene	-	-	0.4
1453	1452	(*E*)-β-Farnesene	0.2	-	-
1455	1454	α-Humulene	2.0	0.5	1.4
1461	1458	*allo*-Aromadendrene	0.5	-	-
1469	1467	9-*epi*-(*E*)-Caryophyllene	0.1	-	-
1476	1478	γ-Muurolene	0.2	-	-
1481	1480	Germacrene D	28.3	4.8	-
1481	1480	γ-Himachalene	-	0.3	0.3
1491	1489	(*Z*,*E*)-α-Farnesene	0.1	-	-
1492	1492	*trans*-Muurola-4(14),5-diene	0.1	-	-
1495	1496	α-Zingiberene	0.2	-	-
1496	1497	Bicyclogermacrene	3.2	0.7	-
1499	1500	α-Muurolene	0.3	-	-
1500	1500	Pentadecane	0.2	-	-
1504	1504	(*E*,*E*)-α-Farnesene	1.0	-	-
1509	1509	β-Curcumene	0.4	-	-
1513	1512	γ-Cadinene	0.2	-	-
1518	1518	δ-Cadinene	1.2	0.5	-
1537	1538	α-Cadinene	tr	-	-
1577	1574	Germacra-1(10),5-dien-4β-ol	0.6	-	-
1583	1587	Caryophyllene oxide	0.4	-	0.3
1604	1607	β-Oplopenone	tr	-	-
1610	1611	Humulene epoxide II	0.2	-	-
1643	1643	τ-Cadinol	0.6	-	-
1645	1645	τ-Muurolol	1.8	-	-
1656	1655	α-Cadinol	0.1	-	-
1738	1735	Mint sulfide	tr	-	-
1833	1835	1-Phytadiene	0.1	-	0.6
1839	1841	Phytone	0.1	-	-
1857	1861	3-Phytadiene	-	-	0.3
1875	1879	4-Phytadiene	-	-	0.2
2144	2143	Serratol	-	1.2	-
2500	2500	Pentacosane	-	1.4	-
		Monoterpene hydrocarbons	tr	0.0	11.7
		Oxygenated monoterpenoids	4.4	9.5	1.2
		Sesquiterpene hydrocarbons	64.4	11.3	6.7
		Oxygenated sesquiterpenoids	3.8	0.0	0.3
		Diterpenoids	0.1	1.2	1.0
		Benzenoid aromatics	1.0	1.3	43.9
		Fatty acid derivatives	26.1	75.3	35.2
		n-Alkanes	0.2	1.4	0.0

Others	0.1	0.0	0.0
Total identified	*99.9*	*100.0*	*100.0*

RI_{calc} = Retention index determined with respect to a homologous series of *n*-alkanes on a ZB-5ms column using the method of van den Dool and Kratz [44]. RI_{db} = Reference retention index obtained from the databases [45–48]. tr = trace (< 0.05%).

Supplementary Table S3. Instrument details for the gas chromatographic analyses of *Scutellaria* species cultivated in Ontario, Oregon.

**Gas Chromatography - Mass Spectrometry (GC-MS)**	
Instrument	Shimadzu GC-MS-QP2010 Ultra (Shimadzu Scientific Instruments, Columbia, MD, USA)
GC Column	Zebron ZB-5ms fused silica capillary column (60 m × 0.25 mm × 0.25 μm film thickness) (Phenomenex, Torrance, CA, USA)
MS Detector Conditions	Electron impact (EI) mode, electron energy = 70 eV, a scan = 40–400 atomic mass units, scan rate = 3.0 scans/second
Carrier Gas, Conditions	Helium, column head pressure = 208.5 kPa, flow rate = 2.00 mL/min
Injector, Detector Temperatures	Injector temperature = 260 °C, interface temperature = 260 °C, ion source temperature = 260 °C
GC Oven Temperature Program	Initial temperature = 50 °C, ramp 2 °C/min to 260 °C, hold 260 °C for 5 min
Sample Concentration, Volume Injected	5% (in dichloromethane), 0.1 μL volume
Split Mode	24.5 : 1.0
**Gas Chromatography - Flame Ionization Detection (GC-FID)**	
Instrument	Shimadzu GC 2010 with FID (Shimadzu Scientific Instruments, Columbia, MD, USA)
GC Column	Zebron ZB-5 GC column (60 m × 0.25 mm × 0.25 μm film thickness) (Phenomenex, Torrance, CA,
Carrier Gas, Conditions	Helium, column head pressure = 208.3 kPa, flow rate = 2.00 mL/min
Injector, Detector Temperatures	260 °C
GC Oven Temperature Program	Initial temperature = 50 °C, ramp 2 °C/min to 260 °C, hold 260 °C for 5 min
Sample Concentration, Volume Injected	5% (in dichloromethane), 0.1 μL volume
Split Mode	24.5 : 1.0
